# Vienna LiverTox Workspace—A Set of Machine Learning Models for Prediction of Interactions Profiles of Small Molecules With Transporters Relevant for Regulatory Agencies

**DOI:** 10.3389/fchem.2019.00899

**Published:** 2020-01-10

**Authors:** Floriane Montanari, Bernhard Knasmüller, Stefan Kohlbacher, Christoph Hillisch, Christine Baierová, Melanie Grandits, Gerhard F. Ecker

**Affiliations:** Pharmacoinformatics Research Group, Department of Pharmaceutical Chemistry, University of Vienna, Vienna, Austria

**Keywords:** Vienna LiverTox Workspace, web service, machine learning, ABC-transporter, OATP-transporter, toxicity, classification models

## Abstract

Transporters expressed in the liver play a major role in drug pharmacokinetics and are a key component of the physiological bile flow. Inhibition of these transporters may lead to drug-drug interactions or even drug-induced liver injury. Therefore, predicting the interaction profile of small molecules with transporters expressed in the liver may help medicinal chemists and toxicologists to prioritize compounds in an early phase of the drug development process. Based on a comprehensive analysis of the data available in the public domain, we developed a set of classification models which allow to predict—for a small molecule—the inhibition of and transport by a set of liver transporters considered to be relevant by FDA, EMA, and the Japanese regulatory agency. The models were validated by cross-validation and external test sets and comprise cross validated balanced accuracies in the range of 0.64–0.88. Finally, models were implemented as an easy to use web-service which is freely available at https://livertox.univie.ac.at.

## Introduction

**Figure 1:**
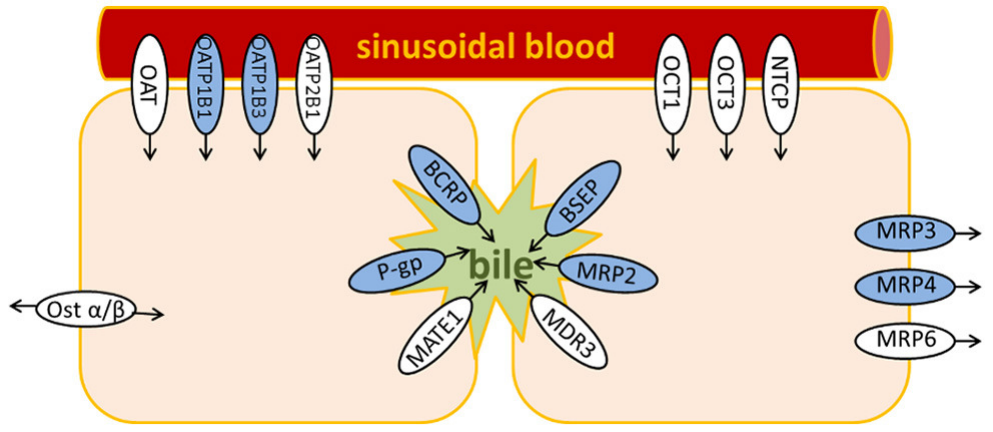
Main hepatic transporters. In blue, transporters for which predictive models (inhibition and/or transport) are available in the Vienna LiverTox Workspace.

Membrane transporters expressed in the liver play different, but interconnected roles: on the one hand, basolateral transporters pick up xenobiotics, and endogenous molecules from the portal vein to the liver, or excrete their substrates into the blood. Apical transporters, on the other hand, take care of the flux toward the bile duct network (Figure 1). Three main types of substrates are of interest with respect to liver toxicity: drugs, which enter the hepatocytes at the first hepatic pass or at the elimination stage; bilirubin, a product of the degradation of the heme; and bile salts, which circulate between the gastro-intestinal tract, and the liver.

Additionally, to the enzyme family of cytochromes, also the transporters expressed in the liver are crucial for a fully functional organ. Some of them are e.g., involved in the bilirubin cycle: OATP1B1 and OATP1B3 uptake bilirubin into the hepatocytes (Briz et al., 2003), where glucuronidation takes place. MRP2 then excretes the bilirubin conjugate to the bile (Kamisako et al., 1999). At the basolateral membrane, MRP3 might also excrete it back to the sinusoidal blood (Keppler, 2014). As a result, inhibition of the uptake OATP transporters or of MRP2 may lead to the accumulation of bilirubin (conjugated or not) in the blood, which is referred to as hyperbilirubinemia. Conjugated hyperbilirubinemia is a marker of hepatobiliary injury (Dufour et al., 2000; Ozer et al., 2008; Padda et al., 2011), and predicting it may allow to flag compounds that could cause liver injury.

Bile acids are synthesized in the liver by catabolism of cholesterol and then excreted to the bile by the active bile salt export pump (BSEP) and by the multidrug resistance-associated protein 2 (MRP2) (Meier and Stieger, 2002). Bile salts have a pronounced detergent effect, which explains their toxicity when they accumulate in the liver (Attili et al., 1986). For their transport in the bile duct, bile salts form mixed micelles with phospholipids of the outer leaflet of the membrane. The multidrug resistance protein 3 (MDR3) allows lipid flopping at the apical membrane of the hepatocyte, and its function is necessary to avoid bile duct toxicity (Nicolaou et al., 2012). After reaching the intestine via the bile flow, bile acids are reabsorbed into the portal vein, and taken up again into the hepatocytes by the sodium taurocholate co-transporting polypeptide (NTCP) (Stieger, 2011). Impairment of bile flow leading to a toxic accumulation of bile salts in the hepatocytes might lead to drug-induced cholestasis, which is one of the main causes of drug-induced liver injury (DILI) (Padda et al., 2011).

Apart from playing a role in proper bile flow and bilirubin elimination, liver transporters also transport drugs that will then be metabolized and excreted. At this stage, drugs can inhibit different transporters and cause drug-drug interactions (König et al., 2013) (in case of co-administered inhibitor and substrate) or liver injury (by disrupting the bile flow for example). This is why predicting the inhibition and the substrate profile for liver transporters might be useful in identifying potentially problematic compounds. In addition, the Food and Drugs administration recommends experimental testing of the interactions between drugs and transporters (especially P-gp, BCRP, OATP1B1, and OATP1B3) to identify potential drug-drug interactions (U.S. Department of Health Human Services Food Drug Administration Center for Drug Evaluation Research, 2012). Thus, it definitely would be of value to have a suite of computational models available which allow the fast and easy assessment of compounds for their interaction profile with transporters expressed in the liver.

Here, we present the Vienna LiverTox Workspace, a web server for the prediction of interactions with liver transporters as well as selected liver toxicity endpoints. To the best of our knowledge, it is the first time that such an ensemble of predictive models for hepatotoxicity and liver transport is made available to the public. The predictions are made by individual machine learning models built on publicly available data for each target of interest.

## Methods

### Data Curation

The datasets for the training and testing of the models were collected from different sources (online tools as well as publications). The data were cleaned by using an in-house system combining the molecular operating environment (MOE 2014.09) (Molecular Operating Environment, 2014) wash option and the Atkinson Standardiser (https://github.com/flatkinson/standardiser). This approach was used for some of the datasets and for others the cleaning procedure of already published papers were used (Pinto et al., 2012; Kotsampasakou et al., 2015). In general, duplicates were removed from the dataset, including pairs of stereoisomers. Further, if these compounds share the same class label, one of the compounds was kept. A detailed list of references as well as the number of compounds revealed after the preprocessing of the data is given in Table S1. In the sections Transporter Models and Hepatotoxicity Models, the data curation as well as the model generation for the specific endpoints is given. The datasets are available in the Supplementary Material (Data Sheet 2).

### Transporter Models

The web service allows for the prediction of interactions between a small molecule and eight different liver transporters (Figure 1, transporters marked in blue). The lack of publicly available data for the other transporters explains the absence of respective models in the Vienna LiverTox Workspace.

All the models predicting whether a compound will be a substrate of a transporter (BCRP, P-gp, BSEP, MRP2, and MRP3) were built upon a dataset correlating expression levels of 47 ABC-transporters with drug toxicity, which can then be used to infer transported vs. non-transported compounds (Szakács et al., 2004). For the transport inhibition models (BCRP, P-gp, BSEP, MRP3, MRP4, OATP1B1, and OATP1B3) the datasets were collected from literature and, if necessary, manually aggregated. In both cases, the models predict a binary outcome: the query compound is a substrate or not, or an inhibitor or not.

For chemistry encoding of the compounds, we used circular fingerprints or 2D molecular descriptors as implemented in RDKit version 2015.03.1 (https://www.rdkit.org/). Different machine learning algorithms were applied and the one giving the best cross-validation results was kept as final model. Especially for the transport prediction, a heavy class imbalancy (most of the drugs in the training set were non-substrates) was noted, which was handled by MetaCost (Domingos, 1999). The exact methodology and cross-validation performance for each individual transporter model is described in the documentation available at https://livertox.univie.ac.at, and an overview is given in Table 1. In some cases, external test sets were collected from Metrabase (Mak et al., 2015) or from recent publications (time-split evaluation).

**Table 1:** Overview of the parameters used for the models.

	**Descriptors**	**Trainings set**	**Algorithm**
**INHIBITION**			
P-gp (MDR1)	ECFP8-like fingerprints	Broccatelli et al., 2011	SVM
BSEP	Molecular descriptors	Warner et al., 2012 Dawson et al., 2012 Morgan et al., 2010	Naïve bayes
BCRP	ECFP8-like fingerprints	Montanari and Ecker, 2014	Logistic regression
MRP3	Molecular descriptors	Köck et al., 2014	BayesNet
MRP4	Molecular descriptors	Köck et al., 2014	AdaBoost (MetaCost)
OATP1B1	Molecular descriptors	De Bruyn et al., 2013	BayesNet
OATP1B3	Molecular descriptors	De Bruyn et al., 2013	BayesNet
**TRANSPORT**			
P-gp (MDR1)	Molecular descriptors	Szakács et al., 2004	Rotation Forest (MetaCost)
BSEP			SVM (MetaCost)
BCRP			k-nearest neighbors (MetaCost)
MRP2			
MRP3			
**TOXICITY**			
Hyperbilirubinemia	ECFP8-like fingerprints	Liu et al., 2011	SVM (MetaCost)
Cholestasis	Molecular descriptors	SIDER v2 database (Kuhn et al., 2010, 2016)	Tree model (MetaCost)
Drug-induced liver injury (DILI)	Molecular descriptors	Various sources^*^	Random Forest

* *see https://livertox.univie.ac.at/ for a detailed list*.

### Hepatotoxicity Models

Three models in the web service can be used to assess human liver damage potentially caused by a test compound: hyperbilirubinemia, cholestasis, and drug-induced liver injury (DILI).

For hyperbilirubinemia, 835 compounds were taken from Kotsampasakou et al. (2017a) and the modeling methodology was kept as in Kotsampasakou et al. (2017b): ECFP-like fingerprints were computed with RDKit (https://www.rdkit.org/), then a combination of feature selection, MetaCost, and support vector machines with RBF kernel was used for learning. The cholestasis model uses data from Kotsampasakou and Ecker (2017) and a combination of MetaCost and a tree algorithm to predict whether a compound is likely to cause cholestasis or not. Finally, the DILI model is based on a 966-compound dataset carefully compiled from literature (Kotsampasakou et al., 2017c). RDKit molecular descriptors and a random forest of 500 trees are used for modeling.

### Web Service Implementation

The Vienna LiverTox Workspace has been implemented as Python/php based web service. It consists of two parts, namely the backend and the frontend. The backend consists of a docker image which runs the machine learning models on an input SD-File. It consists of a Python Flask server (https://palletsprojects.com/p/flask/) which processes the requests from the frontend. Each request consists of one or more input molecules and a list of models to run the predictions on. The frontend, also a docker image, is based on the CakePHP framework (https://cakephp.org/) and is responsible for the user interface (UI), which sends the request to the backend and displays the results. The web service provides, after a login, the possibility to upload a SD-File of 10 compounds. The service can also be used without logging in but then it is only possible to draw and predict a single molecule. JSME (Bienfait and Ertl, 2013) is used as drawing tool. The web service runs on an Ubuntu Linux based server with two twelve-core Intel Xeon 64bit processors and 128 GB RAM, and is hosted at the University of Vienna by the Pharmacoinformatics Research Group.

**Figure 2:**
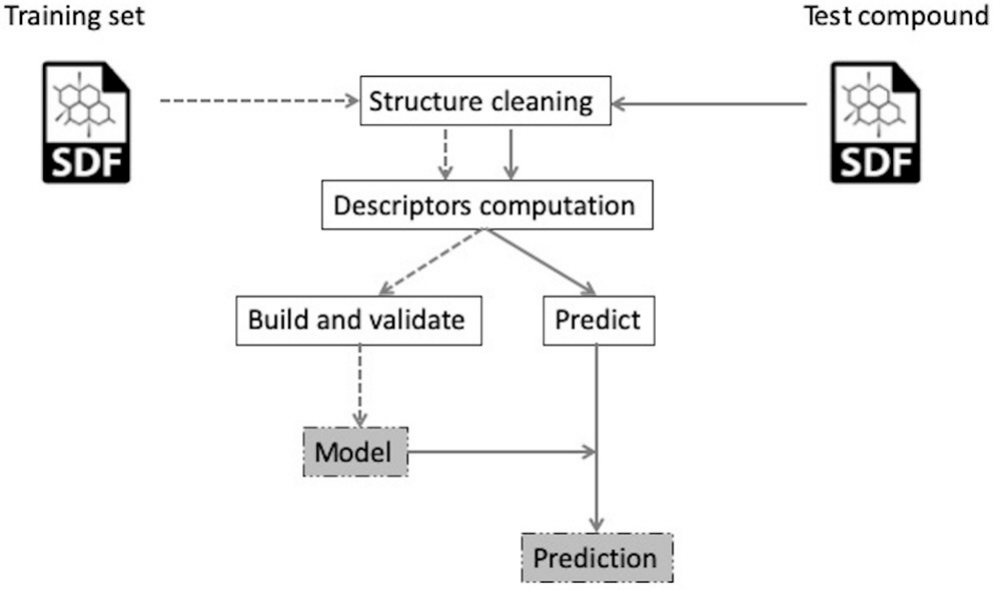
General steps to build a model (left workflow, dotted line) or predict a property for a test compound (right workflow, solid line). Figure adapted from Carrió et al. (2015) published in Journal of Cheminformatics is licensed under CC BY 4.0 (https://creativecommons.org/licenses/by/4.0/).

The models use the RDKit library (https://www.rdkit.org/) (version 2015.03.1) for computing the descriptors and handling the chemistry aspects, while the Weka (Hall et al., 2009) (version 3.7.11) and scikit-learn (Pedregosa et al., 2011) (version 0.14.1) libraries are used to train and run the predictive models. The models also include a compound cleaning step, implemented with the Atkinson Standardiser (https://github.com/flatkinson/standardiser) (Figure 2).

The steps performed during model building and a test compound prediction are shown in Figure 2. In both cases, the compound is standardized and the molecular descriptors are calculated. In the case of the model generation, this allows the training of the model and its development (left workflow). For the prediction of a test compound, the descriptors are passed to the available model to predict its class affiliation (right workflow).

In general, the output of the model gives, in addition to the class prediction, the actual score. This score is a numerical value between 0 and 1, and roughly corresponds to a probability of being active (inhibitor, substrate, or toxic compound). Therefore, a value close to 1 indicates substrate/inhibitor/toxic properties, a value close to 0 annotates for non-substrates/non-inhibitors/non-toxic.

### Applicability Domain

The Applicability Domain (AD) is used to validate the reliability of a given prediction model. It defines whether a dataset of interest is in or out of domain, meaning if it falls within the chemical space of the model or not. If it is out of Domain, the prediction cannot be regarded as reliable.

In our study, an Applicability Domain model, using the approach of Sahigara et al. (2013), was created for each transporter with the respective training set. RDKit descriptors were used as molecule representation. This approach combines the classical, widely used k-Nearest Neighbor (k-NN) method with a probability density function estimation. It uses three stages to determine the reliable space of a prediction model. First, a set of thresholds is defined depending on the diverse densities of the training set by considering the 15 k-nearest neighbors using Euclidean distances. This allows the AD to consider a dense and sparse training region (The threshold defines if a test sample can be reliable predicted). In a next step a decision rule is derived to filter out outlier molecules. Finally, the reliability of the AD is tested by looking at the model statistics and prediction errors. This feature is not yet implemented in the Web service, but will be available soon.

## Results

### Model Results

The performance of the models was estimated by calculating statistical performance metrics using a 10-fold cross-validation. The results are provided in Table 2. The overall accuracy, corresponding to the rate of correct predictions, ranges from 0.59 to 0.87. Also, the sensitivity of the models was calculated (0.57–0.85). This parameter gives the number of actual positives that are correctly identified and is expressed by the number of true positives divided by the number of positive predictions. Further, the number of actual negatives was determined by the number of true negatives divided by the number of negative predictions. The so-called specificity ranges from 0.56 to 0.90. To estimate a metric for the quality of the models, the Matthews correlation coefficient (MCC) and the Area under the Receiver Operating Characteristics curve (ROC AUC) were determined. The MCC is a number between −1 and 1 where 0 indicates a prediction equal to a random prediction and 1 indicates a perfect prediction, whereas −1 is a complete miss. The scores for our models are in the range of 0.20–0.76. The ROC AUC measures the ability of the model to distinguish between negatives and positives, while a higher value indicates a better performance. In the models provided on the Vienna LiverTox Workspace the ROC AUC ranges from 0.64 to 0.94. Furthermore, if data was available, the models were also validated with one or more external test sets. For more details see the documentation on the website.

**Table 2:** Performance metrics of the transporter models.

	**Accuracy**	**Sensitivity**	**Specificity**	**Balanced accuracy**	**MCC**	**ROC AUC**
**INHIBITION**						
P-gp (MDR1)	0.87	0.85	0.90	0.88	0.76	0.94
BSEP	0.85	0.77	0.87	0.82	0.60	0.91
BCRP	0.83	0.77	0.87	0.82	0.64	0.90
MRP3	0.82	0.75	0.87	0.81	0.62	0.86
MRP4	0.73	0.86	0.65	0.76	0.49	0.74
OATP1B1	0.77	0.71	0.78	0.75	0.34	0.80
OATP1B3	0.81	0.78	0.81	0.80	0.35	0.81
**TRANSPORT**						
P-gp (MDR1)	0.81	0.81	0.81	0.81	0.44	0.85
BSEP	0.71	0.65	0.72	0.69	0.28	0.69
BCRP	0.73	0.57	0.75	0.66	0.20	0.71
MRP2	0.70	0.60	0.72	0.66	0.27	0.73
MRP3	0.72	0.60	0.74	0.67	0.28	0.72
**TOXICITY**						
Hyperbilirubinemia	0.67	0.64	0.67	0.66	0.20	0.69
Cholestasis	0.59	0.72	0.56	0.64	0.22	0.64
Drug-induced liver injury (DILI)	0.65	0.72	0.58	0.65	0.30	0.70

### Use Case: Prediction of Liver Interaction for a Propafenone Analog

**Figure 3:**
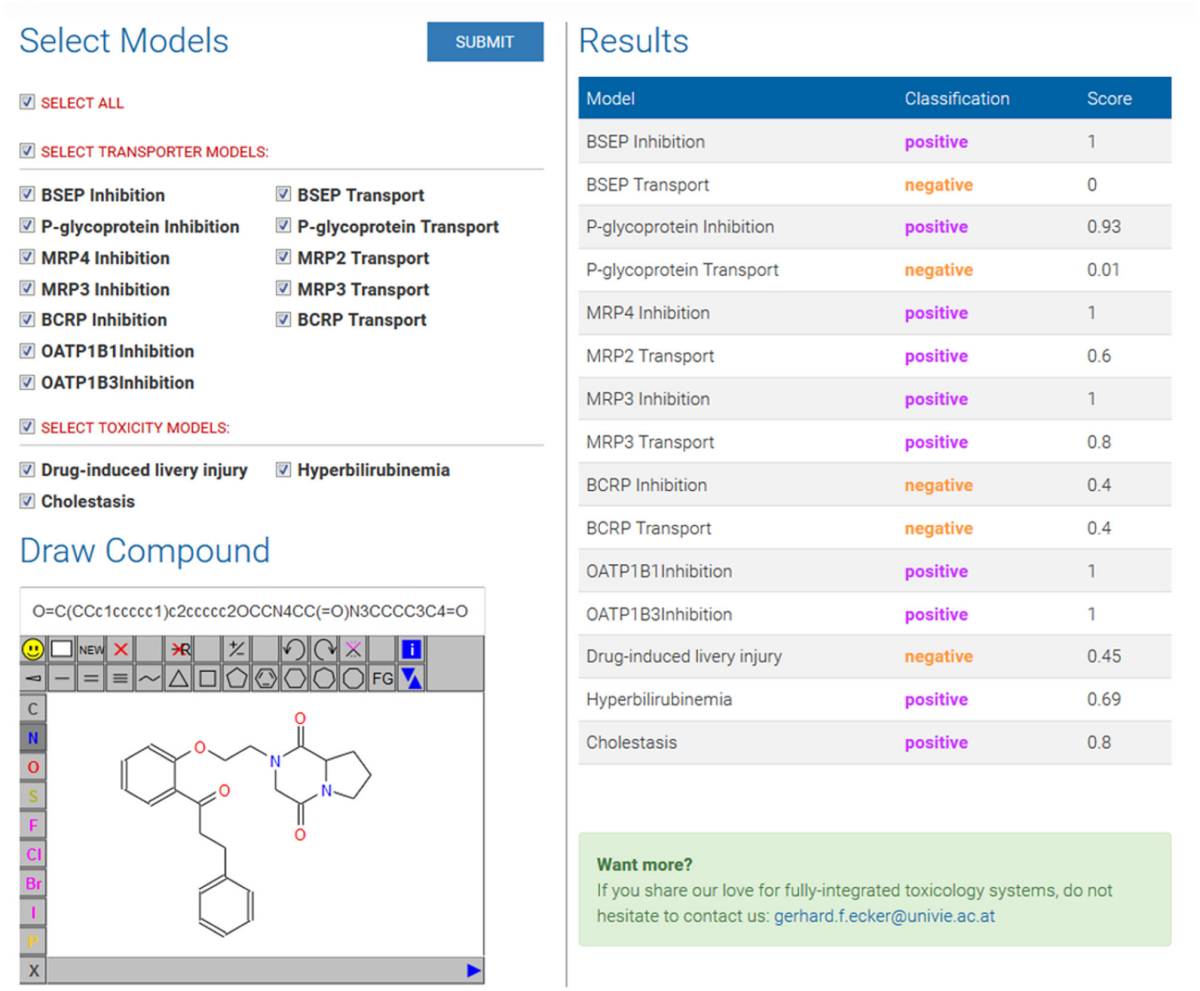
Overview of the web service interface. On the left side, test compound is drawn and desired models are selected. On the right side, results table with the predictions and model scores.

In this section, we briefly detail how predictions can be generated for a given compound using the LiverTox web service. In first instance, the compound is drawn or its SMILES string is pasted in the Molecule Editor (Bienfait and Ertl, 2013). Then the models can be selected on the left panel, either one by one or all at the same time (Figure 3, left side).

By clicking on the SUBMIT button, the data is sent to the backend server where the predictions are running. Upon completion of the calculations, a table listing the different models and corresponding outputs will appear (Figure 3, right side). The second column in the results table corresponds to the binary classification, while the third column “Score” gives the actual output of the model, which corresponds to a probability of being active. For example, for BCRP inhibition and transport and for DILI, the output score is close to 0.5 (which is the threshold used to separate predicted actives from predicted inactives), which indicates an uncertainty of these three models for the particular query compound. Propafenone derivatives are frequently reported as inhibitors of P-gp, and indeed the P-gp inhibition model predicts this particular one to be an inhibitor with a high score (0.93).

## Discussion

Many systems already exist to predict *in silico* activities or properties of small molecules. Table 3 compares freely available ones with our own web service in terms of model offer, submission and run time. For example, ProTox-II predicts oral drug toxicity in rodents (lethal dose LD_50_ and a category of toxicity between 1 and 6) using similarity to compounds with known LD_50_ and recognition of toxic fragments (Drwal et al., 2014). BioZyne proposes exclusively one model for P-gp transport prediction based on the same dataset as ours (Szakács et al., 2004; Levatić et al., 2013). It uses a Support Vector Machine classifier for the prediction of P-gp substrates. The Danish (Q)SAR Database contains pre-calculated properties combined from more than 200 models from both commercial and free tools (http://qsar.food.dtu.dk/). Predictions for environmental toxicity, blood-brain barrier permeation, cytochrome interactions, or human genotoxicity are available. Unfortunately, new predictions for compounds that are not part of the database cannot be made. PkCSM is another web service for predicting pharmacokinetics properties of compounds (Pires et al., 2015). Models such as P-gp inhibition and transport, blood-brain barrier permeation, interaction with cytochromes, renal clearance, or even liver toxicity are available.

**Table 3:** Comparison of existing free online tools to predict ADME-Tox properties of compounds.

**Web service**	**Transporters predictions**	**CYP450 predictions**	**Hepatotox. predictions**	**Batch prediction**	**Run time for 1 compound**
ProTox-II (Drwal et al., 2014)	No	No	No	Yes (max. 100)	<5 s
BioZyne (Levatić et al., 2013)	P-gp	No	No	Not for free	~5 s
QSAR DB (http://qsar.food.dtu.dk/)	No	Yes	No	Yes	N.A.
pkCSM (Pires et al., 2015)	P-gp	Yes	Yes	Yes (max. 100)	<5 s for 30 models
Lazar (Maunz et al., 2013)	No	No	No	No	~10 s for 6 models
Vienna LiverTox Workspace	P-gp, BSEP, BCRP, MRP2, MRP3, MRP4, OATP1B1, OATP1B3	No	Yes	Not for free	~30 s for 15 models

In general, our models for the inhibitors show a better performance especially when looking at the correct prediction of the positives. The prediction of true negatives is for the inhibitor and transporter models quite similar which can be explained by the availability of more negatives if the training set is unbalanced. This is especially the case for the substrate models. The quality of the prediction (MCC) is higher for the inhibition models of P-gp, BSEP, BCRP, and MRP3 since the available dataset is more balanced. In comparison, the three toxicity models show a poorer performance due to the complexity of these endpoints and especially for hyperbilirubinemia and cholestasis which shows also a lack of positives.

The Transporters selected for this web service were chosen based on their importance for regulatory agencies such as FDA, EMA and the Japanese regulatory agency. They recommend or in some cases request these proteins to be routinely tested in inhibition—and substrate studies of new drugs.

## Conclusion

We have presented the Vienna LiverTox Workspace, a web service dedicated to the prediction of liver toxicity and interactions between small molecules and liver transporters. It is easy to use, fast, web browser agnostic, and well-documented. Thanks to its modular system, it will be easy to integrate new models in the future, as well as re-implement existing models in case new training data becomes available. We hope that our models will help researchers to flag potentially dangerous compounds and shed light on the relationships between liver transporters and toxicity.

## Data Availability Statement

All datasets generated for this study are included in the article/Supplementary Material.

## Author Contributions

FM, SK, CH, and MG developed the models. BK, CB, and MG implemented the web service. MG and GE supervised the study. FM and MG wrote the majority of the manuscript. All authors contributed to refining the manuscript.

### Conflict of Interest

The authors declare that the research was conducted in the absence of any commercial or financial relationships that could be construed as a potential conflict of interest.

## Abbreviations

ADME-Tox: absorption distribution metabolism excretion toxicity
BCRP: breast cancer resistance protein
BSEP: bile salt export pump
DILI: drug-induced liver injury
ECFP: extended connectivity fingerprint
MDR1: multidrug resistance protein 1
MDR3: multidrug resistance protein 3
MRP2: multidrug resistance-associated protein 2
MRP3: multidrug resistance-associated protein 3
MRP4: multidrug resistance-associated protein 4
NTCP: sodium taurocholate co-transporting polypeptide
OATP1B1: organic anion transporting polypeptide 1B1
OATP1B3: organic anion transporting polypeptide 1B3
P-gp: P-glycoprotein
SMILES: simplified molecular input line entry system.
