# Supplementary material for: Vienna LiverTox Workspace—A Set of Machine Learning Models for Prediction of Interactions Profiles of Small Molecules With Transporters Relevant for Regulatory Agencies
Source: Front Chem. 2020 Jan 10;7:899. doi: 10.3389/fchem.2019.00899 (PMC6966498; doi:10.3389/fchem.2019.00899)
Supplement: Supplementary file 1 [file Data_Sheet_1.PDF]

Table S1: Sources and Characteristics of the datasets

	Dataset(s)	Cleaning Procedure	Inhibitors/ Substrates/ Toxic Compounds	Non-Inhibitors/ Non-Substrates/ Non-Toxic Compounds	Dataset size (number of compounds)
**Inhibition**					
P-gp (MDR1)					
Training set	(Broccatelli et al., 2011)	(Pinto et al., 2012)	612	549	1161
Test set	(Baumert et al., 2013) (Ochoa-Puentes et al., 2013) (Reis et al., 2013) (Colabufo et al., 2013) (Orlandi et al., 2013) (Contino et al., 2013a) (Contino et al., 2013b) (Capparelli et al., 2014) (Krauze et al., 2014) (Li et al., 2015)	MOE*, Atkinson**	86	48	134
BSEP					
Training set	(Warner et al., 2012) (Dawson et al., 2012) (Morgan et al., 2010)	MOE*, Atkinson**	114	410	524
Test set	(Hirano et al., 2006) (Pedersen et al., 2013)	MOE*, Atkinson**	43	116	159
BCRP					
Training set	(Montanari and Ecker, 2014)	Duplicates removed	432	542	974
Test set	(Contino et al., 2013a) (Winter et al., 2013)	MOE*, Atkinson**	109	86	195

	(Juvale et al., 2013) (Capparelli et al., 2014) (Winter et al., 2014) (Krauze et al., 2014) (Hayashi et al., 2015) (Köhler and Wiese, 2015) (Li et al., 2015)				
MRP3					
Training set	(Köck et al., 2014)	MOE*, Atkinson**	32	52	84
MRP4					
Training set	(Köck et al., 2014)	MOE*, Atkinson**	80	29	51
OATP1B1					
Training set	(De Bruyn et al., 2013)	(Kotsampasakou et al., 2015)	178	1472	1650
Test set	(Karlgren et al., 2012)	(Kotsampasakou et al., 2015)	64	137	201
OATP1B3					
Training set	(De Bruyn et al., 2013)	(Kotsampasakou et al., 2015)	116	1547	1663
Test set	(Karlgren et al., 2012)	(Kotsampasakou et al., 2015)	40	169	209
**Transport**					
P-gp (MDR1)					
Training set	(Szakács et al., 2004)	(Pinto et al., 2012)	123	1067	1190
Test set	Metrabase (Mak et al., 2015)	MOE*, Atkinson**	441	308	749
BSEP					
Training set	(Szakács et al., 2004)	(Pinto et al., 2012)	172	1018	1190
BCRP					
Training set	(Szakács et al., 2004)	(Pinto et al., 2012)	108	1082	1190
Test set	Metrabase (Mak et al., 2015)	MOE*, Atkinson**	246	157	403
MRP2					

Training set	(Szakács et al., 2004)	(Pinto et al., 2012)	247	943	1190
Test set	Metrabase (Mak et al., 2015)	MOE*, Atkinson**	133	118	251
MRP3					
Training set	(Szakács et al., 2004)	(Pinto et al., 2012)	178	1012	1190
Test set	Metrabase (Mak et al., 2015)	MOE*, Atkinson**	58	19	77
**Toxicity**					
Hyperbilirubinemia					
Training set	(Liu et al., 2011)	(Kotsampasakou et al., 2017)	86	749	835
Cholestasis					
Training set	SIDER v2 database (Kuhn et al., 2010, 2016) (Kotsampasakou and Ecker, 2017)	Atkinson**	355	1549	1904
Drug-induced liver injury (DILI)					
Training set	(O'Brien et al., 2006)<br>(Rodgers et al., 2010)<br>(Fourches et al., 2010)<br>(Greene et al., 2010)<br>(Ekins et al., 2010)<br>(Chen et al., 2011)<br>(Liu et al., 2015a)<br>(Zhu and Kruhlak, 2014)<br>(Liu et al., 2015b)	(Kotsampasakou et al., 2015)	500	466	966
Test sets	Mulliner et al., 2016<br>Liew et al., 2011<br>Chen et al., 2016	(Kotsampasakou et al., 2015)	519<br>221<br>50	402<br>120<br>46	921<br>341<br>96

*https://github.com/flatkinson/standardiser ** MOE 2014.09(Molecular Operating Environment, 2014)

References:

Baumert, C., Günthel, M., Krawczyk, S., Hemmer, M., Wersig, T., Langner, A., et al. (2013). Development of small-molecule P-gp inhibitors of the N-benzyl 1,4-dihydropyridine type: Novel aspects in SAR and bioanalytical evaluation of multidrug resistance (MDR) reversal properties. *Bioorganic Med. Chem.* 21, 166–177. doi:10.1016/j.bmc.2012.10.041.

Broccatelli, F., Carosati, E., Neri, A., Frosini, M., Goracci, L., Oprea, T. I., et al. (2011). A Novel Approach for Predicting P-Glycoprotein (ABCB1) Inhibition Using Molecular Interaction Fields. *J. Med. Chem.* 54, 1740–1751. doi:10.1021/jm101421d.

Capparelli, E., Zinzi, L., Cantore, M., Contino, M., Perrone, M. G., Luurtsema, G., et al. (2014). SAR Studies on Tetrahydroisoquinoline Derivatives: The Role of Flexibility and Bioisosterism To Raise Potency and Selectivity toward P-glycoprotein. *J. Med. Chem.* 57, 9983–9994. doi:10.1021/jm501640e.

Chen, M., Vijay, V., Shi, Q., Liu, Z., Fang, H., and Tong, W. (2011). FDA-approved drug labeling for the study of drug-induced liver injury. *Drug Discov. Today* 16, 697–703. doi:10.1016/j.drudis.2011.05.007.

Colabufo, N. A., Contino, M., Cantore, M., Capparelli, E., Perrone, M. G., Cassano, G., et al. (2013). Naphthalenyl derivatives for hitting P-gp/MRP1/BCRP transporters. *Bioorganic Med. Chem.* 21, 1324–1332. doi:10.1016/j.bmc.2012.12.021.

Contino, M., Zinzi, L., Cantore, M., Perrone, M. G., Leopoldo, M., Berardi, F., et al. (2013a). Activity-lipophilicity relationship studies on P-gp ligands designed as simplified tariquidar bulky fragments. *Bioorganic Med. Chem. Lett.* 23, 3728–3731. doi:10.1016/j.bmcl.2013.05.019.

Contino, M., Zinzi, L., Perrone, M. G., Leopoldo, M., Berardi, F., Perrone, R., et al. (2013b). Potent and selective tariquidar bioisosters as potential PET radiotracers for imaging P-gp. *Bioorganic Med. Chem. Lett.* 23, 1370–1374. doi:10.1016/j.bmcl.2012.12.084.

Dawson, S., Stahl, S., Paul, N., Barber, J., and Kenna, J. G. (2012). In vitro inhibition of the bile salt export pump correlates with risk of cholestatic drug-induced liver injury in humans. *Drug Metab. Dispos.* 40, 130–8. doi:10.1124/dmd.111.040758.

De Bruyn, T., van Westen, G. J. P., IJzerman, A. P., Stieger, B., de Witte, P., Augustijns, P. F., et al. (2013). Structure-Based Identification of OATP1B1/3 Inhibitors. *Mol. Pharmacol.* 83, 1257–1267. doi:10.1124/mol.112.084152.

Ekins, S., Williams, A. J., and Xu, J. J. (2010). A predictive ligand-based Bayesian model for human drug-induced liver injury. *Drug Metab. Dispos.* 38, 2302–2308. doi:10.1124/dmd.110.035113.

Fourches, D., Barnes, J. C., Day, N. C., Bradley, P., Reed, J. Z., and Tropsha, A. (2010). Cheminformatics Analysis of Assertions Mined from Literature That Describe Drug-Induced Liver Injury in Different Species. *Chem. Res. Toxicol.* 23, 171–183. doi:10.1021/tx900326k.

Greene, N., Fisk, L., Naven, R. T., Note, R. R., Patel, M. L., and Pelletier, D. J. (2010). Developing Structure−Activity Relationships for the Prediction of Hepatotoxicity. *Chem. Res. Toxicol.* 23, 1215–1222. doi:10.1021/tx1000865.

Hayashi, D., Tsukioka, N., Inoue, Y., Matsubayashi, Y., Iizuka, T., Higuchi, K., et al. (2015). Synthesis and ABCG2 inhibitory evaluation of 5-N-acetylardeemin derivatives the paper is dedicated to Professor Amos B. Smith, III on the occasion of his 70th birthday. *Bioorganic Med. Chem.* 23, 2010–2023. doi:10.1016/j.bmc.2015.03.017.

Hirano, H., Kurata, A., Onishi, Y., Sakurai, A., Saito, H., Nakagawa, H., et al. (2006). High-Speed Screening and QSAR Analysis of Human ATP-Binding Cassette Transporter ABCB11 (Bile Salt Export Pump) To Predict Drug-Induced Intrahepatic Cholestasis.

Mol. Pharm. 3, 252–265. doi:10.1021/mp060004w.

Juvale, K., Stefan, K., and Wiese, M. (2013). Synthesis and biological evaluation of flavones and benzoflavones as inhibitors of BCRP/ABCG2. *Eur. J. Med. Chem.* 67, 115–126. doi:10.1016/j.ejmech.2013.06.035.

Karlgren, M., Vildhede, A., Norinder, U., Wisniewski, J. R., Kimoto, E., Lai, Y., et al. (2012). Classification of inhibitors of hepatic organic anion transporting polypeptides (OATPs): Influence of protein expression on drug-drug interactions. *J. Med. Chem.* 55, 4740–4763. doi:10.1021/jm300212s.

Köck, K., Ferslew, B. C., Netterberg, I., Yang, K., Urban, T. J., Swaan, P. W., et al. (2014). Risk factors for development of cholestatic drug-induced liver injury: inhibition of hepatic basolateral bile acid transporters multidrug resistance-associated proteins 3 and 4. *Drug Metab. Dispos.* 42, 665–74. doi:10.1124/dmd.113.054304.

Köhler, S. C., and Wiese, M. (2015). HM30181 Derivatives as Novel Potent and Selective Inhibitors of the Breast Cancer Resistance Protein (BCRP/ABCG2). *J. Med. Chem.* 58, 3910–3921. doi:10.1021/acs.jmedchem.5b00188.

Kotsampasakou, E., Brenner, S., Jäger, W., and Ecker, G. F. (2015). Identification of Novel Inhibitors of Organic Anion Transporting Polypeptides 1B1 and 1B3 (OATP1B1 and OATP1B3) Using a Consensus Vote of Six Classification Models. *Mol. Pharm.* 12, 4395–4404. doi:10.1021/acs.molpharmaceut.5b00583.

Kotsampasakou, E., and Ecker, G. F. (2017). Predicting Drug-Induced Cholestasis with the Help of Hepatic Transporters—An *in Silico* Modeling Approach. *J. Chem. Inf. Model.* 57, 608–615. doi:10.1021/acs.jcim.6b00518.

Kotsampasakou, E., Escher, S. E., and Ecker, G. F. (2017). Linking organic anion transporting polypeptide 1B1 and 1B3 (OATP1B1 and OATP1B3) interaction profiles to hepatotoxicity - The hyperbilirubinemia use case. *Eur. J. Pharm. Sci.* 100, 9–16. doi:10.1016/j.ejps.2017.01.002.

Krauze, A., Grinberga, S., Krasnova, L., Adlere, I., Sokolova, E., Domracheva, I., et al. (2014). Thieno[2,3-b]pyridines - A new class of multidrug resistance (MDR) modulators. *Bioorganic Med. Chem.* 22, 5860–5870. doi:10.1016/j.bmc.2014.09.023.

Kuhn, M., Campillos, M., Letunic, I., Jensen, L. J., and Bork, P. (2010). A side effect resource to capture phenotypic effects of drugs. *Mol. Syst. Biol.* 6, 343. doi:10.1038/msb.2009.98.

Kuhn, M., Letunic, I., Jensen, L. J., and Bork, P. (2016). The SIDER database of drugs and side effects. *Nucleic Acids Res.* 44, D1075–D1079. doi:10.1093/nar/gkv1075.

Li, X. Q., Wang, L., Lei, Y., Hu, T., Zhang, F. L., Cho, C. H., et al. (2015). Reversal of P-gp and BCRP-mediated MDR by tariquidar derivatives. *Eur. J. Med. Chem.* 101, 560–572. doi:10.1016/j.ejmech.2015.06.049.

Liu, J., Mansouri, K., Judson, R. S., Martin, M. T., Hong, H., Chen, M., et al. (2015a). Predicting Hepatotoxicity Using ToxCast *in Vitro* Bioactivity and Chemical Structure. *Chem. Res. Toxicol.* 28, 738–751. doi:10.1021/tx500501h.

Liu, R., Yu, X., and Wallqvist, A. (2015b). Data-driven identification of structural alerts for mitigating the risk of drug-induced human liver injuries. *J. Cheminform.* 7, 4. doi:10.1186/s13321-015-0053-y.

Liu, Z., Shi, Q., Ding, D., Kelly, R., Fang, H., and Tong, W. (2011). Translating Clinical Findings into Knowledge in Drug Safety Evaluation - Drug Induced Liver Injury Prediction System (DILIps). *PLoS Comput. Biol.* 7, e1002310. doi:10.1371/journal.pcbi.1002310.

Mak, L., Marcus, D., Howlett, A., Yarova, G., Duchateau, G., Klaffke, W., et al. (2015). Metrabase: a cheminformatics and bioinformatics database for small molecule transporter data analysis and (Q)SAR modeling. *J. Cheminform.* 7, 31.

doi:10.1186/s13321-015-0083-5.
Molecular Operating Environment (2014). Available at: https://www.chemcomp.com/.
Montanari, F., and Ecker, G. F. (2014). BCRP Inhibition: from Data Collection to Ligand-Based Modeling. *Mol. Inform.* 33, 322–331. doi:10.1002/minf.201400012.
Morgan, R. E., Trauner, M., van Staden, C. J., Lee, P. H., Ramachandran, B., Eschenberg, M., et al. (2010). Interference with Bile Salt Export Pump Function Is a Susceptibility Factor for Human Liver Injury in Drug Development. *Toxicol. Sci.* 118, 485–500. doi:10.1093/toxsci/kfq269.
O'Brien, P. J., Irwin, W., Diaz, D., Howard-Cofield, E., Krejsa, C. M., Slaughter, M. R., et al. (2006). High concordance of drug-induced human hepatotoxicity with in vitro cytotoxicity measured in a novel cell-based model using high content screening. *Arch. Toxicol.* 80, 580–604. doi:10.1007/s00204-006-0091-3.
Ochoa-Puentes, C., Bauer, S., Kühnle, M., Bernhardt, G., Buschauer, A., and König, B. (2013). Benzanilide–Biphenyl Replacement: A Bioisosteric Approach to Quinoline Carboxamide-Type ABCG2 Modulators. *ACS Med. Chem. Lett.* 4, 393–396. doi:10.1021/ml4000832.
Orlandi, F., Coronnello, M., Bellucci, C., Dei, S., Guandalini, L., Manetti, D., et al. (2013). New structure-activity relationship studies in a series of N,N-bis(cyclohexanol)amine aryl esters as potent reversers of P-glycoprotein-mediated multidrug resistance (MDR). *Bioorganic Med. Chem.* 21, 456–465. doi:10.1016/j.bmc.2012.11.019.
Pedersen, J. M., Matsson, P., Bergström, C. A. S., Hoogstraate, J., Norén, A., LeCluyse, E. L., et al. (2013). Early Identification of Clinically Relevant Drug Interactions With the Human Bile Salt Export Pump (BSEP/ABCB11). *Toxicol. Sci.* 136, 328–343. doi:10.1093/toxsci/kft197.
Pinto, M., Trauner, M., and Ecker, G. F. (2012). An In Silico Classification Model for Putative ABCC2 Substrates. *Mol. Inform.* 31, 547–553. doi:10.1002/minf.201200049.
Reis, M., Ferreira, R. J., Santos, M. M. M., dos Santos, D. J. V. A., Molnár, J., and Ferreira, M.-J. U. (2013). Enhancing Macrocyclic Diterpenes as Multidrug-Resistance Reversers: Structure–Activity Studies on Jolkinol D Derivatives. *J. Med. Chem.* 56, 748–760. doi:10.1021/jm301441w.
Rodgers, A. D., Zhu, H., Fourches, D., Rusyn, I., and Tropsha, A. (2010). Modeling Liver-Related Adverse Effects of Drugs Using *k* Nearest Neighbor Quantitative Structure−Activity Relationship Method. *Chem. Res. Toxicol.* 23, 724–732. doi:10.1021/tx900451r.
Szakács, G., Annereau, J.-P., Lababidi, S., Shankavaram, U., Arciello, A., Bussey, K. J., et al. (2004). Predicting drug sensitivity and resistance: profiling ABC transporter genes in cancer cells. *Cancer Cell* 6, 129–37. doi:10.1016/j.ccr.2004.06.026.
Warner, D. J., Chen, H., Cantin, L.-D., Kenna, J. G., Stahl, S., Walker, C. L., et al. (2012). Mitigating the inhibition of human bile salt export pump by drugs: opportunities provided by physicochemical property modulation, in silico modeling, and structural modification. *Drug Metab. Dispos.* 40, 2332–41. doi:10.1124/dmd.112.047068.
Winter, E., Devantier Neuenfeldt, P., Chiaradia-Delatorre, L. D., Gauthier, C., Yunes, R. A., Nunes, R. J., et al. (2014). Symmetric Bis-chalcones as a New Type of Breast Cancer Resistance Protein Inhibitors with a Mechanism Different from That of Chromones. *J. Med. Chem.* 57, 2930–2941. doi:10.1021/jm401879z.
Winter, E., Lecerf-Schmidt, F., Gozzi, G., Peres, B., Lightbody, M., Gauthier, C., et al. (2013). Structure–Activity Relationships of Chromone Derivatives toward the Mechanism of Interaction with and Inhibition of Breast Cancer Resistance Protein ABCG2. *J. Med. Chem.* 56, 9849–9860. doi:10.1021/jm401649j.
Zhu, X., and Kruhlak, N. L. (2014). Construction and analysis of a human hepatotoxicity

database suitable for QSAR modeling using post-market safety data. *Toxicology* 321, 62–72. doi:10.1016/j.tox.2014.03.009.
